# Rectal metastasis from bladder urothelial carcinoma: a case report

**DOI:** 10.1186/s40792-021-01186-8

**Published:** 2021-04-21

**Authors:** Yuki Ii, Shinya Munakata, Kumpei Honjo, Masaya Kawai, Shingo Kawano, Kiichi Sugimoto, Shuji Isotani, Yutaka Kojima, Shigeo Horie, Takashi Yao, Kazuhiro Sakamoto

**Affiliations:** 1 Department of Coloproctological Surgery, Juntendo University Faculty of Medicine, 2-1-1 Hongo, Bunkyo-ku, Tokyo, 113-8421 Japan; 2 Department of Urological Surgery, Juntendo University Faculty of Medicine, Tokyo, Japan; 3 Department of Human Pathology, Juntendo University Graduate School of Medicine, Tokyo, Japan

**Keywords:** Urothelial carcinoma, Rectal metastasis, Transurethral resection of bladder tumor

## Abstract

**Background:**

Urothelial carcinoma arises from transitional cells in the urothelial tract. In advanced cases, it can metastasize locally to surrounding organs or distally to organs such as the lungs, bones, or liver. Here we describe a case of rectal metastasis from urothelial carcinoma treated with multiple sessions of transurethral resection of bladder tumor (TURBT).

**Case presentation:**

A 72-year-old woman presented to our department with abdominal bloating andobstructed defecation. She had undergone two sessions of TURBT for early urothelial carcinoma in another hospital at 64 and 65 months ago, respectively. Cystoscopy at 3 months after the second TURBT session had indicated disease recurrence, and thus, she had been referred to our hospital for further examination, followed by TURBT for the third time at 59 months ago and for the fourth time at 48 months ago; thereafter, she had been followed up with cystoscopy every 6 months without any recurrence. However, she returned to our hospital, complaining of difficult defecation. Subsequent colonoscopy demonstrated an obstructive tumor in the rectum, which was pathologically diagnosed as metastatic urothelial carcinoma of the bladder. Laparoscopic examination revealed two small areas of peritoneal dissemination in the pelvis. A sigmoid colostomy was performed without rectal tumor resection. She has been receiving chemotherapy and is still alive 10 months after surgery.

**Conclusions:**

Rectal metastasis is a rare site of metastasis for urothelial carcinomas. It is important to consider the possibility of annular rectal constriction caused by infiltrating or metastasizing urothelial carcinoma when managing patients with urothelial carcinoma and with difficult defecation.

## Background

Urothelial carcinoma (UC) is a highly prevalent malignancy of the urinary tract. Therefore, clinicians are considerably familiar with its metastatic sites, which commonly include the bones, lungs, brain, liver, and peritoneum [1]. Rectal metastases of UC are extremely rare, generally occur in cases of advanced bladder cancer, and often indicate a poor prognosis. Furthermore, no standard chemotherapy regimen has been established thus far. While early diagnosis and multimodality therapy result in optimal patient outcomes, metastatic disease is generally incurable, with a relative 5-year overall survival (OS) rate of only 15% [2].

In this report, we present a case of a 72-year-old woman diagnosed with rectal and peritoneal metastases of UC 4 years after the last session of transurethral resection of bladder tumor (TURBT).

## Case presentation

**Fig. 1:**
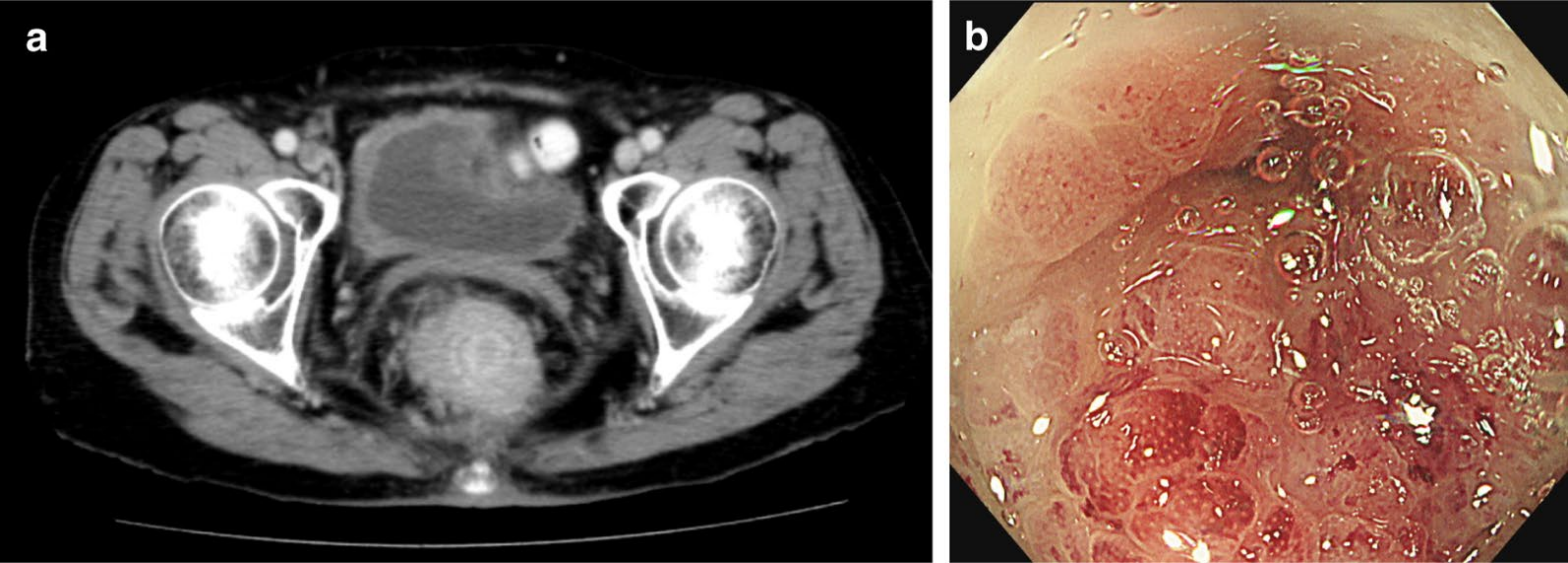
**a** Colonoscopy demonstrated an obstructive tumor in the rectum. **b** CT showed advanced rectal stenosis. CT, computed tomography

The patient was a 72-year-old woman already treated with two sessions of TURBT for pathologically confirmed UC (pTaN0M0, stage 0a) in another hospital at 64 and 65 months ago. BCG treatment was added six times after TURBT.Three months after the second TURBT session, she was referred to our hospital for further examination. Surveillance cystoscopy demonstrated multiple small recurrent papillary tumors in the bladder (diagnosed as pTaN0M0, Grade2, stage 0a), necessitating TURBT for the third and fourth time at 59 and 48 months ago, respectively. Then, she was followed up with cystoscopy every 6 months for 4 years and it showed no disease recurrence,so we weren`t considered of total cystectomy. Five months after the last follow-up cystoscopic examination, which showed no disease recurrence, she presented to our department with chief complaints of difficult defecation and abdominal bloating. Computed tomography (CT) imaging revealed an irregular thickening of the rectal wall without any direct invasion or distant metastases (Fig. 1a).

Colonoscopy demonstrated an obstructive tumor in the rectum, 3–8 cm from the anal verge (Fig. 1b), which was endoscopically biopsied for further examination. The biopsy specimen was found to contain a conglomerate of hyperchromatic and scattered multiangular nuclei. Immunohistochemical analysis revealed that the specimen was positive for cytokeratin (CK)7, CK20, and GATA3 but negative for caudal-type homeobox 2 (CDX2). On cystoscopy, no sign of recurrence was detected in the bladder. Therefore, the rectal lesion was diagnosed as a metastasis of the primary UC. On the basis of these findings, we planned to perform laparoscopic rectal resection. During laparoscopy, two small peritoneal nodules were found in the pelvis, which were subsequently diagnosed as peritoneal metastases of UC by intraoperative frozen section analysis. Hence, we created a loop colostomy in the sigmoid colon without rectal resection.

**Fig. 2:**
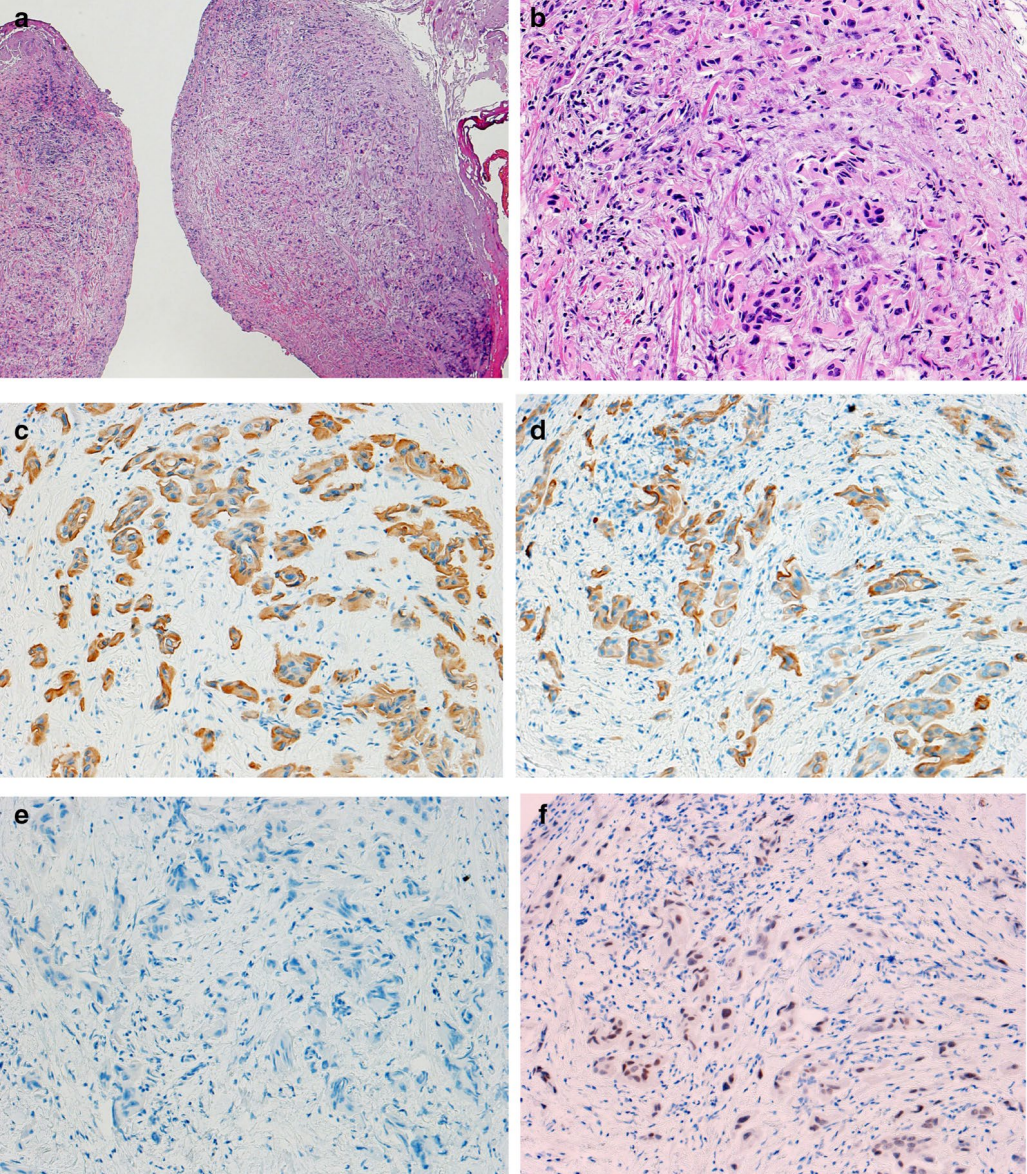
Microscopic examination of the resected specimen revealed a conglomerate of hyperchromatic and scattered multiangular nuclei.. **a** H&E × 40. **b** H&E × 200. Immunohistochemistry showed that the tumor was positive for **c**CK7 (× 200) and **d** CK20 (× 200). **e** Immunohistochemical staining for CDX2 revealed little reactivity (× 200). **f** The tumor appeared diffusely positive for GATA3 (× 200). *H&E* hematoxylin and eosin; *CK* cytokeratin; *CDX2* caudal-type homeobox 2

Histological findings revealed that the pelvic nodules consisted of a conglomerate of both chromatin-concentrated and scattered multiangular nuclei (Fig. 2a and b). They also exhibited the same immunohistochemical staining patterns as did previous TURBT specimens; that is, they appeared positive for CK7, CK20, and GATA3 but negative for CDX2 (Fig. 2c–f). The postoperative course was good and uncomplicated, and the patient's abdominal distention improved following stoma creation. Then, the first cycle of chemotherapy with cisplatin (70 mg/m^2^) plus gemcitabine (1000 mg/m^2^) was initiated on postoperative day (POD) 21. She was finally discharged on POD 38 and later received a total of four chemotherapy cycles as part of an outpatient regimen. Ten months after surgery, the patient remains alive with no evidence of new tumor recurrence other than rectal metastasis.

## Conclusions

Rectal metastases of UC are extremely rare and generally occur in cases of advanced bladder cancer. UC guidelines state that this type of cancer most commonly metastasizes to the lungs, liver, and bone via lymphogenous or hematogenous routes. In a recent review, only a few cases of UC metastases to the colon and rectum were reported [3]. Here, we reported a rare case of rectal metastasis of UC.

Among urogenital cancers, prostate cancer is reported to be the most common cause of rectal obstruction. However, annular constriction of the rectum secondary to bladder cancer has rarely been reported [4]. The mechanism underlying annular rectal stricture caused by UC remains unknown. Yet a number of hypotheses have previously been proposed on how this phenomenon might occur. Stillwell et al. hypothesized that locally aggressive cancer of the bladder neck or trigone might break through Denonvilliers' fascia and encircle the rectum [5]. Langenstroer et al. suggested that surgical deposition of cancer cells might cause rectal obstruction [6]. Kobayashil and Hong et al. attributed annular constriction of the rectum to bladder cancer cell invasion [7, 8].

Kobayashi hypothesized annular rectal obstruction from metastatic bladder cancer was caused of spreading along the lateral pedicles to reach the posterior rectal wall and then infiltrate the rectal wall.

From 1983 to 2020, 10 cases of UC metastasis to the colon (including the present case) have been reported in the literature, with the rectum identified as a site of metastasis in 6 of them (Table 1). Details about the tumor–node–metastasis classification of UC in these cases are not available, but cystectomy represented the most frequent initial or previous treatment [3].

**Table 1:** Metastases of urothelial carinoma to the colorectum

Case	Author	Patient age (years)/sex	TNM *	Initial or previous treatment	Metastatic sites	Treatment after metastasis	Time to metastasis (months)	Outcome
1	Aigen (1983)	77/M	Unknown	Surgery (radical cystectomy)	Sigmoid colon, transverse colon, appendix, cecum, ileum, omentum	Colostomy	198	Unclear [10]
2	Hong (2002)	63/M	Unknown	Surgery (radical cystectomy) Chemotherapy (MVAC)	Rectum and hepatic duct	Radiation therapy	10	Alive (4 months(4 M)) [14]
3	Yusuf (2005)	54/M	Unknown	Surgery (radical cystectomy)	Rectum	Chemotherapy	24	Unclear [15]
4	Yusuf (2005)	73/M	Unknown	Surgery (radical cystectomy)	Rectum	Total pelvic exenteration and chemotherapy	24	Unclear [15]
5	Kakizawa (2006)	57/M	Unknown	Unclear	Sigmoid colon	Unclear	60	Alive (5 M) [16]
6	Chin (2008)	67/M	Unknown	Surgery (partial cystectomy) Chemotherapy (gemcitabine chemotherapy) Radiation therapy	Appendix	Resection of the cecum and terminal ileum, ligation of the right external iliac artery, and end ileostomy	18	Alive (6 M) [17]
7	Kumar (2009)	60/M	Unknown	Surgery (radical cystectomy)	Transverse colon	Unclear	5	Unclear[18]
8	Ying-Yue (2010)	83/M	Unknown	None	Rectum	Chemotherapy	Unclear	Unclear[9]
9	Asfour (2014)	55/M	Unknown	TURBT, 6 weeks Surgery (cystoprostatectomy) Chemotherapy (mitomycin followed by 4 rounds of gemcitabine and cisplatin chemotherapy)	Rectum, omentum, other pelvic structures	Colostomy	15	Brain and lung metastases[19]
10	Our case (2020)	72/M	TaN0M1	TURBT	Rectum, peritoneal nodes	Colostomy and gemcitabine and cisplatin chemotherapy	48	Alive (10 M)

*TNM at initial diagnosis of urothelial carcinoma

The mean time to metastasis was found to be 43.4 (range 5–198) months for nine cases (this variable was unknown for one case). Stoma creation was undertaken as palliative surgery in three cases with annular constriction. Total pelvic exenteration was performed in one case; radiotherapy in one case and chemotherapy in four cases. When treated for the first time, patients in the cases are diagnosed as advanced cancer. Although obstructive defecation was a common complaint, bloody stool was barely reported [3, 9]. Aigen et al. reported a case of UC recurrence after TURBT, which was treated with radiotherapy [10].

Here, we described a rare case of UC (diagnosed as pTaN0M0, stage 0a) recurring despite complete tumor resection in every TURBT session. No direct tumor invasion into the rectal wall and thickened bilateral lateral pedicles were observed on CT imaging, and cystoscopy showed no tumor in the bladder mucosa. Therefore, we postulated that, as suggested by Langenstroer et al., post-TURBT deposition of UC cells might have led to peritoneal dissemination in the pelvis [6].

Accordingly, our patient received the first cycle of chemotherapy on POD 21.

An updated survival analysis of the subjects included in the phase III KEYNOTE-045 trial demonstrated a sustained improvement in the overall survival of patients with recurrent UC 2 years after receiving a second-line treatment with pembrolizumab and thermotherapy [11]. A recent study indicated that first-line pembrolizumab in cisplatin-ineligible patients with advanced UC could elicit clinically enduring responses consistent with those observed in the overall study population [12]. Pembrolizumab is currently the only immune checkpoint inhibitor approved in Japan for the treatment of platinum-refractory UC [13]. Hence, the patient presented here was expected to display improved survival after cisplatin-based chemotherapy.

In conclusion, rectal metastasis represents a rare manifestation of UC and is associated with a poor prognosis. Thus, rectal metastasis of UC should be included as a possibility in the differential diagnosis for obstructive defecation in patients with UC.

## Data Availability

None.

## Abbreviations

UC: Urothelial carcinoma
TURBT: Transurethral resection of bladder tumor
CT: Computed tomography
CK: Cytokeratin
CDX2: Caudal-type homeobox 2
POD: Postoperative day
